# The Impact of Maternal Depression and Parent–Child Interactions on Risk of Parasitic Infections in Early Childhood: A Prospective Cohort in Benin

**DOI:** 10.1007/s10995-021-03317-x

**Published:** 2021-11-30

**Authors:** Amanda Garrison, Joanna Maselko, Marie-Josèphe Saurel-Cubizolles, David Courtin, Roméo Zoumenou, Michael J. Boivin, Achille Massougbodji, André Garcia, Maroufou Jules Alao, Michel Cot, Suzanne Maman, Florence Bodeau-Livinec

**Affiliations:** 1 Département Méthodes Quantitatives en Santé Publique (METIS), Ecole Des Hautes Etudes en Santé Publique, Rennes, France; 2 Center of Research in Epidemiology and Statistics/CRESS, INSERM, INRA, Université de Paris, 75004 Paris, France; 3 Sorbonne Universités, Université de Paris, 75005 Paris, France; 4 Department of Epidemiology, Gillings School of Global Public Health, University of North Carolina, Chapel Hill, NC USA; 5 MERIT, IRD, Université de Paris, Paris, France; 6 Faculté des Sciences de la Santé, Université d’Abomey-Calavi, Cotonou, Bénin; 7 Departments of Psychiatry and Neurology/Ophthalmology, Michigan State University, East Lansing, MI USA; 8 Service de Pédiatrie, CHU de La Mère et de l’Enfant-Lagune de Cotonou, Cotonou, Bénin; 9 Department of Human Behavior, Gillings School of Global Public Health, University of North Carolina, Chapel Hill, NC USA

**Keywords:** Postpartum depression, Parent–child relations, Malaria, Helminths, Child health

## Abstract

**Objectives:**

Maternal depression occurs in 13–20% of women from low-income countries, which is associated with negative child health outcomes, including diarrheal disease. However, few studies have investigated its impact on child risk of infectious disease. We studied the impacts of maternal depressive symptoms and parent–child interactions, independently, on the risk of *Plasmodium falciparum* malaria and soil-transmitted helminth infection in Beninese children.

**Methods:**

Our population included mothers and children enrolled in a clinical trial during pregnancy (MiPPAD) in Benin. The Edinburgh Postnatal Depression Scale (EPDS) assessed maternal depressive symptoms and the home observation measurement of the environment (HOME) assessed parent–child interactions. Blood and stool sample analyses diagnosed child malaria and helminth infection at 12, 18, and 24 months. Negative binomial and Poisson regression models with robust variance tested associations.

**Results:**

Of the 302 mother–child pairs, 39 (12.9%) mothers had depressive symptoms. Median number of malaria episodes per child was 3 (0–14) and 29.1% children had at least one helminth infection. Higher EPDS scores were associated with lower HOME scores; relative risk (RR) 0.97 (95% confidence interval (CI) 0.95, 0.99), particularly with lower acceptance, involvement, and variety subscales; RR 0.92 (95% CI 0.85, 0.99), RR 0.82 (95% CI 0.77, 0.88), RR 0.93 (95% CI 0.88, 0.99), respectively. However, neither exposure was associated with risk of parasitic infection in children.

**Conclusions for Practice:**

Maternal depressive symptoms are associated with poor parent–child interactions, particularly acceptance of behavior, involvement with children, and variety of interactions, but these exposures do not independently impact risk of parasitic infection in children.

## Significance Statement

*What’s already known?* Maternal depression increases risk of child morbidities like diarrheal disease and febrile illness, under the hypothesis that mothers have more difficulty caring for and preventing disease in their children. Previous studies have not considered how these exposures may increase parasitic infectious disease in children living in high-transmission settings.

*What this study adds?* Maternal depressive symptoms impair the way mothers discipline children, interact with them, and provide stimulation and social interactions. Neither maternal depressive symptoms nor impaired parent–child interactions increase the risk of parasitic infection in children in the presence of regular screening and treatment of disease.

## Objectives

Post-natal mental health disorders are a significant public health concern as they have negative consequences not only on maternal health, but also on child well-being and development (Villegas et al., 2011). Women may experience symptoms of maternal depression such as feelings of sadness, tearfulness or hopelessness, loss of energy or appetite, and suicidal ideations from 2 weeks to 12 months after giving birth (Stewart, 2005). Maternal depression affects 13–20% of women globally, according to the World Health Organization (WHO) (World Health Organization, n.d.). Women in developing countries, such as Sub-Saharan African (SSA) countries, are at higher risk of developing depression after pregnancy due to their lower socioeconomic status, lower social support, reduced access to mental health services, and higher rates of maternal morbidities compared to women in higher income countries (Sawyer et al., 2010). However, diagnosis of maternal depression and measurement of associated symptoms remains widely underestimated in many SSA countries, including in Benin, due to a lack of validated tools for diagnosis.

Maternal depression is linked to several consequences on child health and development, such as stunting and underweight (Avan et al., 2010). A meta-analysis in 2017 concluded that maternal depressive symptoms are also associated with lower cognitive scores in infancy (Liu et al., 2017). Depressed mothers may interact differently with their infants compared to non-depressed mothers; they may be more withdrawn and provide less stimulation for children necessary for neurocognitive and psychological development (Azak & Raeder, 2013). Poor parent–child interactions in the home environment are thought to be the mediator between maternal depression and child cognitive development (Bass et al., 2016). Few studies have investigated how maternal depression and parent–child interactions independently impact child morbidity from infectious disease. One study found that post-natal depression increases risk of diarrheal illness in one-year-old children in a low-income setting (Rahman et al., 2007). Another linked post-natal depression to increased risk of febrile illness in Ghanaian and Ivorian infants one year after birth (Guo et al., 2013).

There are several hypothesized mechanisms for the relationship between maternal depression and child morbidity, including the role of various biological, hormonal, cultural, and social factors (Coughlin, 2012; Weinstock, 2005). Authors of previous studies hypothesized that maternal depression could impact a mother’s ability to complete daily activities, care for, and provide support for her child, therefore putting them at higher risk of various morbidities, including otherwise preventable infectious diseases (Patel et al., 2002). However, there are currently no existing studies, to our knowledge, on the impact of parent–child interactions on child parasitic infection.

In Benin, malaria caused by the *Plasmodium falciparum* parasite is the leading cause of mortality in children under five years (President’s Malaria Initiative, n.d.). Infections from soil-transmitted helminths due to insufficient hygiene and sanitation systems also contribute to the burden of disease in this part of the world, with one study showing a prevalence of 23% in a population of children in Benin (Ibikounle et al., 2018). These parasitic infections have serious consequences for child health and are leading causes of anemia in children in SSA (Mulu et al., 2014). However, despite the increased interest in studies investigating the links between mental and physical health in mothers and children, no such studies exist on the impact of maternal depression, or maternal depressive symptoms, and parent–child interactions on parasitic infection in early childhood. Similar to the hypothesis made by Rahman and colleagues (Rahman et al., 2007), we hypothesized that women suffering from symptoms of maternal depression, and women who interact with and stimulate their children less, may also have more difficulties caring for their children and providing them with necessary preventive measures against parasitic infection, therefore resulting in higher risk of malaria and helminth infection. The aims of this paper were to study the impacts of maternal depressive symptoms and parent–child interactions, independently, measured at one year postpartum on the risk of *P. falciparum* malaria and soil-transmitted helminth infections in children from a Beninese cohort.

## Methods

### Cohort Selection

Our study population included women and their children who participated in the malaria in pregnancy preventive alternative drugs (MiPPAD) clinical trial comparing the efficacy of sulfadoxine-pyrimethamine and mefloquine used for IPTp in women (NCT0081142). Further information on the study protocol and inclusion criteria for this clinical trial may be found in a previous publication (González et al., 2014). The trial took place in the semi-rural area of Allada, about 40 km outside of Cotonou, Benin, in Sub-Saharan Africa. All eligible, liveborn singletons of women were invited to participate in a follow-up study (TOVI) at 12 months of age, whose aim was to study associations between maternal anemia and neurodevelopment in children (Bodeau-Livinec et al., 2016). A sub-cohort of these children was then selected to participate in the prospective study immune tolerance associated with malaria: consequences for the protection of pregnant women and children (TOLIMMUNPAL), which followed children from 12 to 24 months to measure *P. falciparum* malaria and soil-transmitted helminth infection.

### Maternal Depressive Symptoms and Parent–Child Interactions

Maternal depressive symptoms was assessed in women by trained investigators during a home visit one year postpartum through the Edinburgh Postnatal Depression Scale (EPDS), a 10-item Likert-style questionnaire used to measure symptoms of depression by inquiring about feelings of sadness or hopelessness, changes in appetite or activity, and ideations of self-harm within the previous seven days. The French version of the EPDS was translated by local investigators into Fon, the language spoken in Allada. The EPDS score was recorded and analyzed as a continuous score, with higher scores indicating more depressive symptoms. A pilot study validated the French-translated EPDS for use in this setting, given that there are no gold standard clinical diagnostic tests for maternal depression in Benin, the details for which can be found in a previous publication (Koura et al., 2013). Parent–child interactions were also assessed by trained investigators during this visit through the Home Observation Measurement of the Environment (HOME), which was adapted for use in this setting during the same pilot study. The HOME test is a combination of interview questions and observations of parent–child interactions in the home environment and consists of several subscales. An example of an adaptation made to the version of the HOME used in our population included the removal of one question asking how much time the child spends outside, as children in this population spend most of their time outside of the home. The six sub-scales included in the adapted version of the HOME test were responsivity (verbal interactions between parent and child), acceptance (how the parent disciplines the child), organization (how the home and child’s personal space is organized), learning materials (toys and activities available for child development), involvement (interactions between parent and child), and variety (opportunities for variety and social interactions in the child’s daily routine).

### Parasitic Infection

The main outcomes of interest in our study, *P.falciparum* malaria and soil-transmitted helminth infection, were repeatedly measured in children from 12 to 24 months of age through the collection and analysis of blood and stool samples. Malaria was diagnosed by a Rapid Diagnostic Test (RDT) and/or a thick blood smear test administered systematically in children bi-weekly during the follow-up period as well as during emergency visits to a health clinic if children presented with fever (≥ 37.5 °C). Helminth infection was measured at 12, 18, and 24 months in children, using the Kato-Katz technique on a thick smear of stool sample (World Health Organization, 1994). Children found to be positive for either malaria or helminth infections were subsequently treated within the confines of the study; adherence to helminth prophylaxis was not directly monitored by researchers.

### Covariates

Socio-demographic information was collected through a questionnaire given to women during the home visit one year post-partum. Maternal age, education, and marital status were noted. A family possession score was generated to determine socio-economic status; this score was comprised from a list of possessions owned by the family such as a motorcycle, a car, two cows, a bicycle, and electricity. Household size, the number of people living in the same house as the child, was noted. Women were asked about their overall hygiene practices, hand-washing habits, and accessibility to latrines in a transversal study (Study of birthweight as a predictive factor for child health status; EPOPEE); this information was used to create a synthesized hygiene rating using Multiple Correspondence Analysis (MCA) as outlined by Cortinovis et al (1993). Environmental risk of malaria was calculated as a time- and space-dependent assessment of environmental risk of infection that quantified each child’s exposure to malaria vectors using a predictive model (Lysaniuk et al., 2015). Environmental risk was log-transformed for statistical analyses. During each visit, parents were asked if their child had slept under a mosquito-net the previous night; children were then classified as sleeping under a mosquito net always, frequently, occasionally, or never according to the proportion of times their parents answered “yes” to the question. Participating children also had available information on malaria episodes prior to 12 months.

### Statistical Analysis

Characteristics of our population to the population of children excluded from the 12-month parasitic infection follow-up. Univariate analyses investigated associations of potential confounding factors identified in direct acyclic graphs (DAGs). Linear regression models tested associations between maternal depressive symptoms and the HOME score, both measured cross-sectionally at 12-months post-partum, controlling for socio-economic status in the form of a family possession score, maternal education, and marital status (Patel et al., 2002; Stein et al., 1991). Negative binomial and logistic regression models tested associations between main exposures measured at 12 months and parasitic outcomes measured longitudinally from 12 to 24 months. These models controlled for factors that were significant in univariate and final models. All regression models were derived using generalized linear models to generate log-linear models, and therefore effect estimates are presented as relative risk (RR) with a 95% confidence interval (CI) based on outputs. Statistical analyses were completed using STATA 13.1 (StataCorp. 2013. Stata Statistical Software: Release 13. College Station, TX: StataCorp LP), with an alpha risk of 5%.

### Sensitivity Analyses

Missing data for mosquito net use and hygiene rating were 6% and 18%, respectively; therefore, models with and without these potential confounders were carried out. Malaria was tested in additional models as a binary variable, each HOME sub-scale was tested for potential associations with parasitic infection, and number of malaria in children before 12 months of age (yes/no) was considered in additional models.

### Ethical Approval

The institutional review boards of the Hospital Clinic of Barcelona, the Comité Consultatif de Déontologie et d’Éthique of the Institut de Recherche pour le Développement, the University of Abomey-Calavi, New York University (IRB#09-1253), and the Beninese Ethical Committee of the Faculté des Sciences de la Santé (FSS) approved all study protocols included in this paper. Written informed consent of women was obtained in the presence of a witness, with thumbprints provided if women could not read and/or write.

## Results

**Fig. 1:**
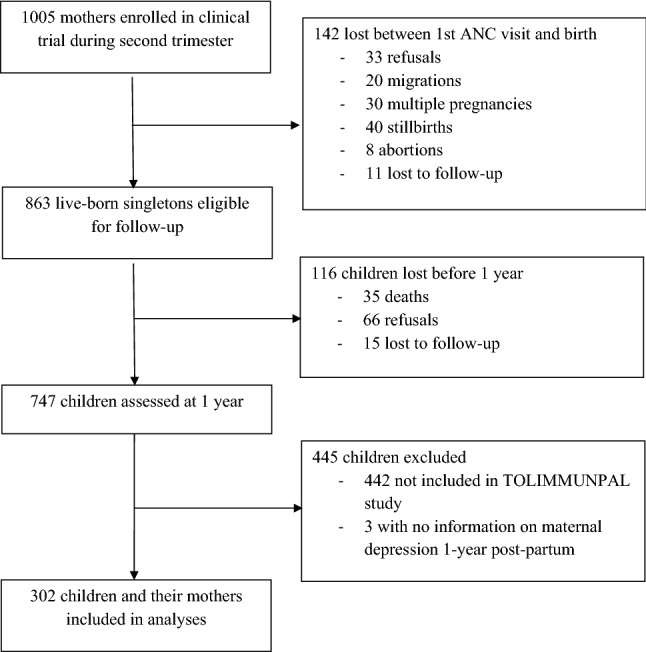
Population flowchart of follow-up from pregnancy to one year of age in children

Of the 1005 pregnant women recruited into the MiPPAD clinical trial, 863 singleton children were born alive to these women [Fig. 1]. One-year post-partum, 747 mother–child pairs were followed up and assessed for maternal depressive symptoms and parent–child interactions. Our study included a sub-population of these children, 302 of whom were recruited for the TOLIMMUNPAL study to assess parasitic infection from 12 to 24 months of age and included in our analyses.

Socio-demographic characteristics of our population are displayed in Table 1, along with characteristics of mother–child pairs not included in the 12-month prospective follow-up for parasitic infection. Mean maternal age of our population was 27 years, 39.4% of women had some education, and 63.3% of women were in monogamous marriages. Mean HOME score among women and children was 26.6. Using a cut-off score of 13, 12.9% of participating women showed symptoms of maternal depression according to the EPDS.

**Table 1:** Socio-demographic characteristics of mother–child pairs included and excluded from prospective follow-up in TOLIMMUNPAL

Parameter	Category	Included (N = 302) N (%) or mean (SD^a^)	Excluded (N = 442) N (%) or mean (SD)	P-value^b^
Maternal education	None	183/302 (60.6)	270/437 (61.8)	0.74
	Some	119/302 (39.4)	167/437 (38.2)	
Maternal age (years)		27.3 (6.1)	26.7 (5.6)	0.28
Maternal Body Mass Index (BMI)	Underweight	19/302 (6.3)	22/329 (6.7)	0.06
	Normal	248/302 (82.1)	247/329 (75.1)	
	Overweight/obese	35/302 (11.6)	60/329 (18.2)	
Family possession score quartile^c^	1st	89/302 (29.5)	113/437 (25.9)	0.17
	2nd	97/302 (32.1)	120/437 (27.5)	
	3rd	93/302 (30.8)	166/437 (38.0)	
	4th	23/302 (7.6)	38/437 (8.7)	
Household size (number of people)		5.0 (1.6)	4.9 (1.6)	0.60
Marital status	Monogamous	190/300 (63.3)	273/430 (63.5)	0.97
	Polygamous	110/300 (36.7)	157/430 (36.5)	
Parity	Primipara	47/302 (15.6)	69/329 (21.0)	0.08
	Multipara	255/302 (84.4)	260/329 (79.0)	
Child sex	Male	146/302 (48.3)	232/442 (52.5)	0.27
	Female	156/302 (51.7)	210/442 (47.5)	
Birth weight	Normal (≥ 2500 g)	277/300 (92.3)	283/320 (88.4)	0.10
	Low (< 2500 g)	23/300 (7.7)	37/320 (11.6)	
Gestational age (ballard score)		38.0 (2.8)	38.5 (3.5)	0.11
Child iron deficiency	No	157/296 (53.0)	241/366 (65.9)	0.001*
	Yes	139/296 (47.0)	125/366 (34.1)	
HOME score		26.6 (2.4)	27.0 (2.2)	0.14
Depressive symptoms (EPDS)	< 13	263/302 (87.1)	370/436 (84.9)	0.40
	≥ 13	39/302 (12.9)	66/436 (15.1)	

^a^SD = standard deviation

^b^Fisher-exact test for categorical variables, Wilcoxon rank-sum test for continuous variables

^c^1st quartile refers to group with fewest possessions (i.e. most deprived), 4th quartile refers to group with most possessions (i.e. least deprived)

*P ≤ 0.05

During the 12-month follow-up for parasitic infection in children, 84.1% of children had at least one malaria episode [Table 2]. The average number of malaria episodes per child was 3, ranging from 0 to 14 episodes per child. Of the children assessed for helminth infection, 29.1% had at least one infection. At 12 and 18 months, the majority of helminth infection were caused by *Trichuris trchiura* species, (73.9%) and (51.3%) respectively. At 24 months, 73.6% of infections were caused by hookworm species.

**Table 2:** Malaria and soil-transmitted helminth infection in children from 12 to 24 months of age

Infection	N (%)
Number of malaria episodes	
None	48/302 (15.9)
1	49/302 (16.2)
2	59/302 (19.5)
3 +	146/302 (48.3)
Average number of episodes per child^a^	3 (0–14)
Average parasite density (parasites/µL)^a^	100,036 (0–869,071)
Malaria before 12 months	87/302 (29)
Number of helminth infections	
None	200/282 (70.9)
1	72/282 (25.5)
2	10/282 (3.6)
Helminth infection at 12 months	23/223 (10.3)
*Ankylostomes*	2/23 (8.7)
*Ascaris lumbricoides*	2/23 (8.7)
*Trichuris trichiura*	17/23 (73.9)
*Schistosoma mansoni*	1/23 (4.3)
Other (*Enterobius vermicularis*)	1/23 (4.3)
Helminth infection at 18 months	39/200 (19.5)
*Ankylostomes*	8/39 (20.5)
*A. lumbricoides*	13/39 (33.3)
*T. trichiura*	20/39 (51.3)
*S. mansoni*	0
Other	0
Helminth infection at 24 months	30/197 (15.2)
*Ankylostomes*	8/30 (26.8)
*A. lumbricoides*	14/30 (46.8)
*T. trichiura*	10/30 (33.4)
*S. mansoni*	0
Other	0

^a^Mean (range)

Univariate analyses between potential confounders and outcomes are displayed in Table 3. No maternal education, lower family possession score, normal birthweight, higher environmental risk of malaria, and previous history of malaria were risk factors for higher incidence of malaria episodes in children. Helminth infection in children was associated with higher maternal age, multiparae mothers, higher maternal BMI, and more people living in the household.

**Table 3:** Univariate analyses between potential confounding factors and parasitic infection outcomes

Variable	Missing N (%)	Malaria between 12 and 24 months		Helminth infection between 12 and 24 months		
		Episodes (median)	P-value^a^	Not infected N (%)	Infected N (%)	P-value^b^
Maternal age	0		0.32			0.05*
≥ 25		2		111 (76)	35 (24)	
> 25		3		89 (65)	47 (35)	
Maternal education	0		0.08*			0.82
None		3		119 (70)	50 (30)	
Some		2		81 (72)	32 (28)	
Maternal BMI	0		0.81			0.08*
Underweight		3		15 (94)	1 (6)	
Normal		2		159 (69)	73 (31)	
Overweight/obese		2		26 (76)	26 (24)	
Family possession score (quartile)	0		0.21			0.55
1st		2		66 (77)	20 (23)	
2nd		3		62 (68)	29 (32)	
3rd		2		28 (70)	12 (30)	
4th		2		44 (68)	21 (32)	
Household size	0		0.43			0.08*
≤ 5		2.5		132 (75)	45 (25)	
> 5		2		68 (65)	37 (35)	
Marital status	0		0.33			0.89
Monogamous		3		75 (71)	30 (29)	
Polygamous		2		125 (71)	52 (29)	
Parity	0		0.26			0.01*
Primipara		2		38 (86)	6 (14)	
Multipara		3		162 (68)	76 (32)	
Child sex	0		0.86			0.70
Male		2		95 (70)	41 (30)	
Female		3		105 (72)	41 (28)	
Birth weight	2 (< 1)		0.004*			0.36
Normal (≥ 2500 g)		2		183 (70)	77 (30)	
Low (< 2500 g)		1		16 (80)	4 (20)	
Gestational age			0.79			0.46
Term (≥ 37 weeks)		2		177 (70)	75 (30)	
Preterm (< 37 weeks)		3		23 (77)	7 (23)	
Iron deficient	6 (2)		0.70			0.55
No		3		108 (73)	40 (27)	
Yes		2		92 (70)	40 (30)	
Environmental risk^c^	5 (2)	0.12	0.04*	–	–	–
Mosquito net use	19 (6)		0.85			–
Never/occasionally		3			–	
Frequently		2			–	
Always		3			–	
Malaria before 1 year			0.01*			–
No		2			–	
Yes		3			–	
Hygiene rating	54 (18)		–			0.78
Inferior		–		27 (73)	10 (27)	
Average		–		73 (68)	35 (32)	
Superior		–		64 (67)	32 (33)	

^a^P-value of Wilcoxon rank-sum test (for two-category variables) or Kruskal–Wallis test (for more-than-two-category variables)

^b^P-value of chi-squared test

^c^Spearman’s rank correlation coefficient for two continuous variables

*P ≤ 0.20

Symptoms of maternal depression were associated with poorer parent–child interactions measured by the HOME test RR 0.98 (95% CI 0.96, 0.99) [Table 4], after verifying linearity between the two variables. Higher EPDS score was associated with lower acceptance of child misbehavior RR 0.91 (95% CI 0.85, 0.99), lower parental involvement with the child at home RR 0.82 (95% CI 0.77, 0.87) and less variety of daily stimulation and social interaction provided for the child RR 0.93 (95% CI 0.88, 0.98). These associations remained in models adjusted for significant confounders within univariate analyses.

**Table 4:** Associations between maternal depression and parent–child interactions one-year post-partum

	Maternal depression	
	Model 1^a^ N = 302	Model 2^b^ N = 302
HOME total score	0.98 (0.96, 0.99)*	0.97 (0.95, 0.99)*
Responsivity	0.99 (0.95, 1.02)	0.97 (0.93, 1.01)
Acceptance	0.91 (0.85, 0.99)*	0.92 (0.85, 0.99)*
Organization	1.03 (0.95, 1.11)	1.02 (0.94, 1.10)
Learning materials	1.02 (0.97, 1.07)	1.01 (0.95, 1.07)
Involvement	0.82 (0.77, 0.87)*	0.82 (0.77, 0.88)*
Variety	0.93 (0.88, 0.98)*	0.93 (0.88, 0.99)*

^a^Crude RR (95% CI)

^b^Adjusted RR (95% CI) for socio-economic variables: family possession score, maternal education, marital status

*P ≤ 0.05

Crude and adjusted negative binomial regression models did not reveal associations between maternal depressive symptoms and total malaria episodes in children RR 1.00 (95% CI 0.97, 1.03) and RR 1.00 (95% CI 0.98, 1.03), respectively; nor between maternal depressive symptoms and helminth infection RR 0.99 (95% CI 0.95, 1.04) and RR 1.00 (95% CI 0.95, 1.05), respectively [Table 5]. Crude and adjusted logistic regression models between maternal depressive symptoms and parent–child interactions were also found to have no association with helminth infection in children. Mosquito-net use and hygiene rating were kept in final models, despite not being associated with outcomes and not significantly changing effect estimates of final models if excluded, due to being important protective factors against parasitic infection (ter Kuile et al., 2003; Vaz Nery et al., 2019).

**Table 5:** Associations between maternal depression and parent–child interactions exposures and child parasitic infection outcomes

	Maternal depression RR (95% CI)	Parent–child interactions RR (95% CI)
Total malaria episodes		
Crude^a^	1.00 (0.97, 1.03)	0.97 (0.94, 1.01)
Adjusted^b^	1.00 (0.98, 1.03)	0.98 (0.94, 1.02)
Soil-transmitted helminth infection		
Crude^c^	0.99 (0.95, 1.04)	1.05 (0.96, 1.15)
Adjusted^d^	1.00 (0.95, 1.05)	1.02 (0.93, 1.12)

^a^N = 302

^b^Adjusted for maternal education, family possession score, household size, environmental risk, low birthweight, mosquito-net use, and history of malaria; N = 281

^c^N = 282

^d^Adjusted for maternal age and hygiene rating; N = 250

*P ≤ 0.05

## Conclusions for Practice

Our hypothesis that women with more depressive symptoms and worse parent–child interactions would be associated with higher risk of parasitic infection in children was not confirmed within analyses. While maternal depressive symptoms are associated with worse parent–child interactions, this does not seem to impact risk of malaria and soil-transmitted helminth infection in children.

Strengths of our prospective cohort study include the repeated biological assessments for malaria and helminth infection, along with anthropological and neurodevelopmental assessments, which few African cohorts have done before. Two diagnostic techniques were used to assess malaria, with the thick blood smear test being the gold standard of malaria diagnosis according to the WHO. Another strength of this study is the consideration of several potential confounding factors identified in the literature, such as socio-economic status, household size, the use of mosquito bed nets, and hygiene habits, including hand-washing. Sensitivity analyses were completed using a binary outcome for malaria during the 12-month follow-up and no associations were found between exposures and malaria.

A limitation of this study is the low power due to small sample size; malaria-helminth co-infection could not be analyzed in this study due to the low number of co-infections. Selection bias due to attrition could also be present; however, maternal depressive symptoms and parent–child interactions did not significantly differ between our population and the mother–child pairs excluded from TOLIMMUNPAL. Our population of 302 children were more iron deficient (47%) compared to excluded children (34.1%). Mothers in our population could have been depressed before one-year post-partum, however only parasitic infection diagnosed after administration of the EPDS was considered for temporality. Most infants in high malaria-transmission settings experience their first malaria episodes several months or one year after birth due to acquired immunity (Dobbs & Dent, 2016). Our study controlled for children who had malaria episodes before the onset of the study period, which increases their risk for subsequent malaria episodes.

Our study shows that maternal depressive symptoms impact how mothers are able to care and provide for their children; however, in the presence of close screening and treatment of parasitic infection these factors do not increase risk of parasitic infection in children. If malaria or helminth infection was diagnosed in children, treatment was given to them during the visit to the health clinic, therefore potentially underestimating the effect of depressive symptoms and parent–child interactions on parasitic infection. The study by Guo et al., which found maternal depression to increase risk of febrile illness in children, collected the physical status of children at just two timepoints: 3 months and 12 months of age (Guo et al., 2013). Our results suggest that in the presence of more regular screening and treatment of parasitic infection in children, maternal depressive symptoms and parent–child interactions do not impact risk of subsequent infection.

About 13% of women in our population were identified as having symptoms of maternal depression one-year post-partum according to a cut-off score of 13, similar to studies from other SSA cohorts using the same cut-off (Ebeigbe & Akhigbe, 2008; Okronipa et al., 2012). Our analyses also support findings that show maternal depressive symptoms are associated with poor parent–child interactions after controlling for socio-economic risk factors. The link between post-partum depression and impaired mother–child interactions has been established in previous works (Gueron-Sela et al., 2018; Lefkovics et al., 2014; Stein et al., 1991), with many researchers hypothesizing that this association is a mediator for poor neurodevelopmental outcomes in children. One study found that treatment of post-partum depression in mothers improved the quality of mother–child interactions and of infant play (Goodman et al., 2008). Our study adds to this literature by further exploring how maternal depressive symptoms may impact specific aspects of parent–child interactions measured by the HOME sub-scales. Our results show that maternal depressive symptoms negatively impact the way mothers discipline their children, physically interact with them, and provide variety in stimulation and social interactions outside the home. Other studies have concluded that poor physical interactions between depressed mothers and their children impair the intellectual stimulation needed by children for proper cognitive and emotional development at a young age (Azak & Raeder, 2013). Within the pilot study, the HOME was also found to be associated with maternal education and cognitive development measured by the Mullen Scales of Early Learning (MSEL) in children at 1 year of age (Koura et al., 2013), as seen in a previous study in Uganda (Bass et al., 2016).

Few previous studies have explored the relationship between maternal depression and child morbidity (Guo et al., 2013; Rahman et al., 2007). Authors have hypothesized that socio-economic status and education level are common risk factors for both and could play important roles in this mechanism (Familiar et al., 2016), which is why family possession score of families and maternal education were included in our analyses. It has also been hypothesized that maternal depression, which can lead to hormonal abnormalities, could compromise the immune system and subsequent response to viral infection (Schuster et al., 2011). While this mechanism was not explored in this study in the context of parasitic infection, future studies could further elaborate on this pathway. Maternal depression can also make it difficult for mothers to perform daily tasks and chores (Rahman et al., 2007), including providing adequate care for their children. In terms of disease treatment and prevention, mothers with depression were found to have more medication nonadherence for treatment of human immunodeficiency virus (HIV), compared to non-depressed mothers (Schuster et al., 2011).

Despite current prevention and treatment recommendations, our study shows a high proportion of malaria (84.1%) and soil-transmitted helminth (29.1%) infection at least once in children during the 12-month follow-up. While 75% of our population reported always sleeping under ITNs at night, there is a potential for social desirability bias which could overestimate the true frequency of ITN-use for malaria prevention in households. Of the children who were infected by helminths between 12 and 24 months, 87.6% of them were only infected once, indicating that there was very little chronic infection in children and that once children were treated, most remained uninfected.

Our study considered maternal depressive symptoms and parent–child interactions separately in their role on child parasitic infection, however future studies could further explore the mediating factor of parent–child interactions in the association between maternal depression, or maternal depressive symptoms, and child morbidity in larger prospective cohorts in order to have more statistical power. *P. falciparum* malaria and soil-transmitted helminth infection carry a large burden of disease in children in Benin, as seen by the large prevalence of infection in our population. Adequate screening, prevention, and treatment must be available for at risk groups such as children under the age of five. Further studies carried out in Benin and other SSA countries could also seek to measure prevalence of maternal depression to add to the limited existing literature.

The associations found within this study between maternal depression and parent–child interactions reiterate the need for adequate mental health interventions and follow-up in low-resource countries like Benin where maternal depression and its symptoms can have potential long-term consequences on child health and development. A qualitative study is currently underway in this population to complement the quantitative results on the impacts of maternal depression on child parasitic infection and provide valuable data to better understand the experiences and barriers to adequate preventive treatment for parasitic infection during pregnancy.

## References

[CR1] Avan B, Richter LM, Ramchandani PG, Norris SA, Stein A (2010). Maternal postnatal depression and children’s growth and behaviour during the early years of life: Exploring the interaction between physical and mental health. Archives of Disease in Childhood.

[CR2] Azak S, Raeder S (2013). Trajectories of parenting behavior and maternal depression. Infant Behavior and Development.

[CR3] Bass JK, Nakasujja N, Familiar-Lopez I, Sikorskii A, Murray SM, Opoka R, Augustinavicius J, Boivin MJ (2016). Association of caregiver quality of care with neurocognitive outcomes in HIV-affected children aged 2–5 years in Uganda. AIDS Care.

[CR4] Benin | PMI. (n.d.). Retrieved May 4, 2018, from https://www.pmi.gov/where-we-work/benin

[CR5] Bodeau-Livinec F, Glorennec P, Cot M, Dumas P, Durand S, Massougbodji A, Ayotte P, Le Bot B (2016). Elevated blood lead levels in infants and mothers in benin and potential sources of exposure. International Journal of Environmental Research and Public Health.

[CR6] Cortinovis I, Vella V, Ndiku J (1993). Construction of a socio-economic index to facilitate analysis of health data in developing countries. Social Science & Medicine.

[CR7] Coughlin SS (2012). Anxiety and depression: Linkages with viral diseases. Public Health Reviews.

[CR8] Dobbs KR, Dent AE (2016). Plasmodium malaria and antimalarial antibodies in the first year of life. Parasitology.

[CR9] Ebeigbe PN, Akhigbe KO (2008). Incidence and associated risk factors of postpartum depression in a tertiary hospital in Nigeria. The Nigerian Postgraduate Medical Journal.

[CR10] Familiar I, Murray S, Ruisenor-Escudero H, Sikorskii A, Nakasujja N, Boivin MJ, Opoka R, Bass JK (2016). Socio-demographic correlates of depression and anxiety among female caregivers living with HIV in rural Uganda. AIDS Care.

[CR11] González R, Desai M, Macete E, Ouma P, Kakolwa MA, Abdulla S, Aponte JJ, Bulo H, Kabanywanyi AM, Katana A, Maculuve S, Mayor A, Nhacolo A, Otieno K, Pahlavan G, Rupérez M, Sevene E, Slutsker L, Vala A, Menéndez C (2014). Intermittent preventive treatment of malaria in pregnancy with mefloquine in HIV-infected women receiving cotrimoxazole prophylaxis: A multicenter randomized placebo-controlled trial. PLoS Medicine.

[CR12] Goodman SH, Broth MR, Hall CM, Stowe ZN (2008). Treatment of postpartum depression in mothers: Secondary benefits to the infants. Infant Mental Health Journal.

[CR13] Gueron-Sela N, Camerota M, Willoughby MT, Vernon-Feagans L, Cox MJ, Family Life Project Key Investigators (2018). Maternal depressive symptoms, mother-child interactions, and children’s executive function. Developmental Psychology.

[CR14] Guo N, Bindt C, Te Bonle M, Appiah-Poku J, Hinz R, Barthel D, Koffi M, Posdzich S, Deymann S, Barkmann C, Schlüter L, Jaeger A, Blay Nguah S, Eberhardt KA, N’Goran E, Tagbor H, Ehrhardt S, International CDS Study Group (2013). Association of antepartum and postpartum depression in Ghanaian and Ivorian women with febrile illness in their offspring: A prospective birth cohort study. American Journal of Epidemiology.

[CR15] Ibikounle M, Onzo-Aboki A, Doritchamou J, Tougoue J-J, Boko PM, Savassi BS, Siko EJ, Dare A, Batcho W, Massougbodji A, Kinde-Gazard DA, Kabore A (2018). Results of the first mapping of soil-transmitted helminths in Benin: Evidence of countrywide hookworm predominance. Plos Neglected Tropical Diseases.

[CR16] Koura KG, Boivin MJ, Davidson LL, Ouédraogo S, Zoumenou R, Alao MJ, Garcia A, Massougbodji A, Cot M, Bodeau-Livinec F (2013). Usefulness of child development assessments for low-resource settings in francophone Africa. Journal of Developmental and Behavioral Pediatrics.

[CR17] Lefkovics E, Baji I, Rigó J (2014). Impact of maternal depression on pregnancies and on early attachment. Infant Mental Health Journal.

[CR18] Liu Y, Kaaya S, Chai J, McCoy DC, Surkan PJ, Black MM, Sutter-Dallay A-L, Verdoux H, Smith-Fawzi MC (2017). Maternal depressive symptoms and early childhood cognitive development: A meta-analysis. Psychological Medicine.

[CR19] Lysaniuk B, Ladsous R, Tabeaud M, Cottrell G, Pennetier C, Garcia A (2015). Types of homes and ways of life: A territorial analysis of the environmental determinants that factor into the proliferation of malaria vectors in the rural region of Allada in Benin. Rural and Remote Health.

[CR20] Mulu A, Kassu A, Legesse M, Erko B, Nigussie D, Shimelis T, Belyhun Y, Moges B, Ota F, Elias D (2014). Helminths and malaria co-infections are associated with elevated serum IgE. Parasites & Vectors.

[CR21] Okronipa HET, Marquis GS, Lartey A, Brakohiapa L, Perez-Escamilla R, Mazur RE (2012). Postnatal depression symptoms are associated with increased diarrhea among infants of HIV-positive Ghanaian mothers. AIDS and Behavior.

[CR23] Patel V, Rodrigues M, DeSouza N (2002). Gender, poverty, and postnatal depression: A study of mothers in Goa, India. The American Journal of Psychiatry.

[CR24] Rahman A, Bunn J, Lovel H, Creed F (2007). Maternal depression increases infant risk of diarrhoeal illness: A cohort study. Archives of Disease in Childhood.

[CR25] Sawyer A, Ayers S, Smith H (2010). Pre- and postnatal psychological wellbeing in Africa: A systematic review. Journal of Affective Disorders.

[CR26] Schuster R, Bornovalova M, Hunt E (2011). The influence of depression on the progression of HIV: Direct and indirect effects. Behavior Modification.

[CR27] Stein A, Gath DH, Bucher J, Bond A, Day A, Cooper PJ (1991). The relationship between post-natal depression and mother-child interaction. The British Journal of Psychiatry.

[CR28] Stewart D (2005). Depression during pregnancy. Canadian Family Physician.

[CR29] ter Kuile FO, Terlouw DJ, Kariuki SK, Phillips-Howard PA, Mirel LB, Hawley WA, Friedman JF, Shi YP, Kolczak MS, Lal AA, Vulule JM, Nahlen BL (2003). Impact of permethrin-treated bed nets on malaria, anemia, and growth in infants in an area of intense perennial malaria transmission in western Kenya. The American Journal of Tropical Medicine and Hygiene.

[CR30] Vaz Nery S, Pickering AJ, Abate E, Asmare A, Barrett L, Benjamin-Chung J, Bundy DAP, Clasen T, Clements ACA, Colford JM, Ercumen A, Crowley S, Cumming O, Freeman MC, Haque R, Mengistu B, Oswald WE, Pullan RL, Oliveira RG, Brooker SJ (2019). The role of water, sanitation and hygiene interventions in reducing soil-transmitted helminths: Interpreting the evidence and identifying next steps. Parasites & Vectors.

[CR31] Villegas L, McKay K, Dennis C-L, Ross LE (2011). Postpartum depression among rural women from developed and developing countries: A systematic review. The Journal of Rural Health.

[CR32] Weinstock M (2005). The potential influence of maternal stress hormones on development and mental health of the offspring. Brain, Behavior, and Immunity.

[CR22] World Health Organization. (1994). *Bench aids for the diagnosis of intestinal parasites*. World Health Organization. Retrieved May 9, 2019 from https://apps.who.int/iris/handle/10665/37323

[CR33] WHO | Maternal mental health. (n.d.). WHO; World Health Organization. Retrieved February 27, 2020, from https://www.who.int/mental_health/maternal-child/maternal_mental_health/en/

